# Plant Essential Oil with Biological Activity

**DOI:** 10.3390/plants11070980

**Published:** 2022-04-04

**Authors:** Hazem S. Elshafie

**Affiliations:** School of Agricultural, Forestry, Food and Environmental Sciences, University of Basilicata, Via dell’Ateneo Lucano 10, 85100 Potenza, Italy; hazem.elshafie@unibas.it; Tel.: +39-0971-205522; Fax: +39-0971-205503

**Keywords:** natural products, antimicrobial activity, cytotoxicity, phytotoxicity, antioxidant, phytopathogens, food preservatives

## Abstract

Plant essential oils (PEOs), extracted from many aromatic and medicinal plants, are used in folk medicine and often represent an important part of the traditional pharmacopoeia: they have a long history of use in folk medicine as antimicrobial agents to control several human and phyto-pathogens. Many PEOs have been registered as effective alternatives to chemical and synthetic antimicrobials, and in the last few decades, they have also been effectively used in the food industry as antioxidants and anticarcinogens, thanks to the efforts of many research/medical institutions and pharmaceutical companies. This Special Issue discussed the chemical composition and biological-pharmaceutical activities of some important PEOs and their single constituents. Detailed information has been also covered in this Special Issue regarding the mechanisms, possible modes of action, and factors affecting these activities, such as geographical origins, environmental conditions, nutritional status, and the extraction methods used.

## 1. Introduction

This Special Issue entitled ‘Plant Essential Oil with Biological Activity’ comprises 15 papers covering a wide range of different aspects, ranging from the biochemical characterization of some PEOs and their biopharmaceutical activities and possible agri-food and medical applications. In particular, some important published research papers have manipulated different aspects related to the biological investigation of crude essential oils (EOs), whereas other papers were related to the study of their specific single constituents. Some other research papers focused on the comparative chemical profiles of different species/ecospecies of the same plant. In addition, some studies have highlighted the seasonal changes in the production and utilization stages of some PEOs. Furthermore, some research studies are related to the use of EOs for the control of serious phytopathogens. On the other hand, our Special Issue has also covered some important review papers covering different aspects of bio-pharmaceutical properties and agri-food applications.

## 2. Antimicrobial, Phytotoxic Activity, and Agri-Food Applications

Recently, the use of some EOs as alternative antimicrobial and pharmaceutical agents has attracted considerable interest from scientists worldwide [1]. In particular, Abd-ElGawad et al. [2] reviewed the phytotoxic effect of some EOs and their chemical compositions as reported in bibliographic research from 1972 to 2020. The same authors used chemometric analysis to build a structure–activity relationship between phytotoxicity and EO chemical composition. In particular, the analysis of the collected data revealed that oxygenated terpenes and mono- and sesquiterpenes play principal roles in the phytotoxicity of EOs [2].

Another important study, conducted by Abd-ElGawad et al. [3], deals with the chemical composition of EO extracted from the aerial parts of *Persicaria lapathifolia* and its free radical scavenging activity and its herbicidal effect on the weed *Echinochloa colona*. The results obtained showed that the extracted EO of *P. lapathifolia* exhibited substantial allelopathic activity against the germination, seedling root, and shoot growth of the weed *E. colona* in a dose-dependent manner [3]. In addition, *P. lapathifolia* EO demonstrated promising antioxidant activity [3]. On the other hand, Abd-ElGawad et al. [4] evaluated the phytotoxic activity of EOs extracted from two ecospecies of *Pulicaria undulata* (Saudi and Egyptian) against the weeds *Dactyloctenium aegyptium* and *Bidens pilosa*. The results obtained showed that the EO of the Egyptian ecospecies showed more phytotoxic activity against *D. aegyptium* and *B. pilosa* than the Saudi ecospecies [4].

An important study conducted by Ibáñez and Blázquez [5] reviewed the agri-food applications of *Curcuma longa* L. rhizome EO. This review study focused on some interesting information regarding conventional and recent extraction methods of *C. longa* rhizome oil, their characteristics, and their suitability to be applied at the industrial scale [5].

An important study published in this Special Issue, conducted by Xylia et al. [6], evaluated the efficacy of an eco-friendly product based on rosemary and eucalyptus Eos and two different application methods (vapor and dipping) on the quality attributes of tomato fruits, and the obtained results indicated that eco-friendly products based on the studied EOs were able to maintain the quality of tomato fruits. In addition, Chrysargyris et al. [7] studied the vapor application of sage EO for maintaining tomato fruit, where the quality attributes were more affected in green fruits and were less affected red fruits. The results also showed that sage EO has a lowering effect of the total phenolics, acidity, total soluble solids, and fruit chroma, with no specific trend found in both breaker and red tomatoes.

Calvopiña et al. [8] studied the chemical analysis of a new sesquiterpene essential oil from the native Andean species *Jungia rugosa* and studied its cholinergic activity. The results showed that the volatile fraction of this EO was exclusively composed of sesquiterpenes, specially curcumene (more than 45%) and sesquiphellandrene (about 17%). This EO demonstrated weak inhibition activity against AChE.

Regarding the antimicrobial activity, Camele et al. [9] studied the potential microbicide activity of *Mentha piperita* cv. ‘Kristinka’ and its main constituents against some common phytopathogens (*Botrytis cinerea*, *Monilinia fructicola*, *Penicillium expansum,* and *Aspergillus niger*). The results obtained showed that the tested EO has promising antifungal activity against all tested fungi. In addition, Soliman et al. [10] studied the antifungal activity of *Mentha spicata* and *Mentha longifolia* EOs against *F. oxysporum*. The results obtained also showed that the single compounds (thymol, adapic acid, menthol, and menthyl acetate) possess antifungal effects through the malformation and degradation of the fungal cell wall [10].

## 3. Bio-Pharmaceutical Properties

Many plant EOs are being widely utilized in the pharmaceutical industry, aromatherapy, and other related medical applications. Regarding the cytotoxicity effect and antioxidant activity, several studies have been published in this Special Issue. Elgamal et al. [11] studied the chemical profiles of EOs of the above-ground parts of *Pluchea dioscoridis* (L.) DC. and *Erigeron bonariensis* (L.) in addition to their cytotoxic and anti-aging activities. The results obtained explicated that the terpenoids are the main constituents of both plants, with a relative concentration of 93.59% and 97.66%, respectively, mainly including sesquiterpenes (93.40% and 81.06%) [11]. Another study conducted by Shahin et al. [12] reported that the flowers’ EO extracted from *Aerva javanica*, isolated during four seasons, is considered a good source of natural bioactive antioxidants. Khalil et al. [13] studied the chemical composition of EO isolated from *Anisosciadium lanatum* and evaluated its anti-cancer potential and mechanistic effect on HepG2 liver cancer cell lines. The obtained results showed that the studied EO was able to regulate the cell proliferation and cell viability in HepG2 liver cancer cells at a sub-lethal dose of 10 to 25 g/mL and displayed reductions in migration and invasion [13]. In addition, the treatment with *A. lanatum* EO indicated the mitigation of cancer activity by aborting the mRNA of pro-apoptotic markers such as BCL-2,CASPASE-3, CYP-1A1, and NF_k_B [13].

## 4. Future Perspectives

Further studies are required to determine the consumer demand for agri-food product-based EOs; however, a balance must be struck between matching customer expectations and maximizing industrial production efficiency in accordance with Green Chemistry. On the other hand, reality simulation models are required in order to determine the composition of essential oils based on the environmental conditions surrounding plants; these models should aim to manage these variables and thus obtain high yield oils with the appropriate chemical composition for specific functions in the agri-food sector. In addition, the use of PEOs as food preservatives should be investigated further to determine the optimum application regarding the effective dose, the time of exposure, and the number of treatments. Regarding the herbicidal effect of PEOs, further future studies are recommended in order to characterize the allelochemical substances of many EOs against various weeds. In the same context, additional investigations are also needed to encapsulate EOs to maximize their biological activities and to examine the application of single active ingredients for several purposes.

## 5. Conclusions

As conclusion, the studies in this Special Issue prove that several studied PEOs have promising pharmaceutical, medicinal, and culinary benefits. In addition, other studies highlighted the potential applications of numerous EOs in the agri-food industry, where they have a strong antimicrobial activity against a broad spectrum of food spoilage microorganisms and extend food shelf-lives. Furthermore, the studied EOs and their two main constituents can be used successfully as possible natural alternatives to synthetic substances against several phytopathogens. Many researchers have also attributed the biological activity of many EOs to the characteristic chemical composition, usually sesquiterpenes and phenolic compounds. We hope that we were able to contribute to the enrichment of the scientific content of the reader in this important field of research.
